# Quantifying Eligibility Pattern Shifts: a Data-Driven Paradigm for Early Risk Detection in Clinical Trials

**DOI:** 10.1007/s43441-026-00918-y

**Published:** 2026-02-06

**Authors:** Atanu Bhattacharjee, Ayon Mukherjee

**Affiliations:** 1 Division of Population Health and Genomics, University of Dundee, Dundee, United Kingdom; 2 Population Health Sciences Institute, Newcastle University, Newcastle, United Kingdom

**Keywords:** Risk-Based Monitoring (RBM), Centralized Monitoring, Eligibility Heterogeneity, Site-Level Risk Assessment, Bayesian Monitoring Framework, Enrollment Pattern Shift, Baseline Inclusion Criteria, Clinical Trial Quality Assurance, Adaptive Oversight, Shiny Decision-Support Tool

## Abstract

Traditional Risk-Based Monitoring (RBM) strategies emphasise key risk indicators and site-level performance metrics but seldom address the heterogeneity of patient eligibility profiles. We present a data-driven framework that captures temporal and inter-site shifts in baseline inclusion characteristics. Central to this framework are two new metrics-*Borderline Inclusion Index* and *Eligibility Distribution Divergence*-that quantify departures from expected enrolment patterns. A Bayesian composite score synthesises these indicators to prioritise oversight actions. Through simulation experiments and a worked case study, we show that monitoring eligibility pattern shifts offers an early warning signal of operational or scientific risk and strengthens overall trial integrity. We operationalize the framework through an interactive Shiny web application that computes indicator-specific posteriors, generates composite site risk scores, and provides visual decision-support for centralized RBM implementation.

## Introduction

Risk-Based Monitoring (RBM) has emerged as a key innovation in clinical trial oversight, aiming to ensure participant safety, data integrity, and operational efficiency while reducing the burden of traditional on-site monitoring [1, 2]. The shift from exhaustive source data verification (SDV) to RBM reflects the recognition that risks to trial validity are not uniformly distributed across sites, participants, or data domains [3, 4]. Instead, RBM emphasizes proactive identification of critical-to-quality factors and targeted monitoring strategies.

Several empirical studies and methodological frameworks have demonstrated the effectiveness of RBM in reducing monitoring costs and focusing attention on sites with higher risk profiles. For example, centralized statistical monitoring has been shown to detect data anomalies, fraud, or unusual patterns more efficiently than routine visits [5, 6]. Similarly, adaptive risk indicators, including adverse event reporting rates, protocol deviations, and enrollment speed, have been incorporated into RBM dashboards [7, 8]. Despite these advances, one dimension of trial conduct remains underexplored: the variability in how enrollment criteria are applied across sites.

Eligibility criteria are fundamental to defining the study population and ensuring both internal validity and external generalizability. However, in multicenter trials, heterogeneity in the interpretation or operationalization of enrollment thresholds (e.g., laboratory cutoffs, comorbidity exclusions, performance status definitions) can introduce systematic biases and safety concerns [9, 10]. For instance, excessive leniency in borderline cases may inflate event rates, while overly stringent application may restrict recruitment and undermine representativeness.

The role of enrollment criteria variability as a risk signal in RBM has not been systematically addressed in the literature. Existing monitoring frameworks tend to focus on site performance metrics or post-enrollment data quality, rather than proactively auditing the front end of the trial-the process by which patients are selected into the study [11]. We argue that enrollment patterns themselves, when systematically quantified, constitute a valuable source of risk information. By incorporating statistical measures of distributional heterogeneity (e.g., site-level vs. trial-wide distributions of age, laboratory markers, comorbidity prevalence), monitoring teams can identify atypical sites earlier and intervene before downstream data integrity or safety issues arise.

In this paper, we propose a methodological framework for RBM that integrates enrollment criteria variability into centralized monitoring systems. The framework provides tools for quantifying heterogeneity in eligibility distributions, developing site-level risk scores, and dynamically updating monitoring strategies. In doing so, it aligns with regulatory emphasis on quality-by-design principles and strengthens the methodological foundations of RBM.

## Background and Methodological Framework

Risk-Based Monitoring (RBM) has become central to modern trial oversight, reflecting regulatory imperatives to embed quality-by-design principles into study conduct [1, 2, 12]. While RBM has reduced reliance on exhaustive source data verification and improved detection of anomalous site performance [3, 5, 6], its current indicators remain primarily operational or post-enrollment in nature. This creates a critical blind spot: the enrollment process itself, which encompasses pre-screening, screening, post-screening follow-up, and adherence to protocol-defined window periods, is rarely monitored quantitatively despite being a key determinant of internal validity and generalizability [9–11].

### Methodological Challenges Across Enrollment Stages

At the **pre-screening** stage, potential participants are initially assessed against broad eligibility criteria. Variability arises when sites apply differing thresholds for borderline cases or use inconsistent laboratory assays. Pre-screening logs often reveal substantial heterogeneity in the proportion of patients deemed ineligible, but these data are seldom integrated into monitoring frameworks. At the **screening** stage, when detailed assessments are conducted, challenges include protocol deviations in laboratory timing, inconsistent application of comorbidity exclusions, and differing interpretations of performance status. Sites under recruitment pressure may be more likely to admit participants whose laboratory values sit at or just beyond the thresholds. The **post-screening** stage, covering the period between eligibility confirmation and randomization, is also at risk: delays in re-checking time-sensitive parameters (e.g., hematology or biochemistry) may allow patients whose values subsequently drift outside the window to be enrolled, undermining the integrity of eligibility rules. Finally, **window periods** defined in protocols-for example, requiring that laboratory tests be performed within 14 days of randomization-introduce further complexity. Sites may differ in their adherence to these temporal windows, leading to hidden heterogeneity in baseline characteristics and potentially biasing time-to-event outcomes.

### The Role of Data Distributions

These enrollment-related risks are best understood not simply as binary deviations, but as distributional phenomena. For example, if creatinine clearance must exceed 60 mL/min, the distribution of this measure among enrolled participants should be relatively similar across sites once random variability is accounted for. A site whose distribution is markedly shifted toward the threshold, or that admits an unusually high proportion of borderline cases, signals a systemic risk. Likewise, screen-failure distributions provide insight: abrupt shifts in the frequency of specific failure reasons (e.g., liver function abnormalities, ECOG performance status) may indicate operational drift or inconsistent application of eligibility rules. Modeling these phenomena requires methods that can capture both central tendency and distributional tails. Classical tools such as Kolmogorov–Smirnov tests can highlight gross divergences, while modern approaches such as energy distance (a metric sensitive to differences in means, variances, and shapes [13]) or hierarchical Bayesian shrinkage models provide stability for smaller sites and sensitivity to subtle distributional shifts [5, 6].

### Enrollment Process Metrics and their Distributional Importance

In addition to eligibility-based indicators, risk-based monitoring must account for operational metrics that describe the tempo and quality of enrollment processes themselves. These metrics, when analyzed across sites, yield distributional patterns that are highly informative for identifying latent risks.

*Screening velocity*. The speed with which sites move potential participants through the screening process is a critical marker of operational quality. Extremely rapid screening may suggest superficial assessments or overlooked eligibility checks, whereas unusually slow screening can reflect logistical barriers, staffing constraints, or difficulty in accessing eligible populations. The distribution of screening times across sites thus serves as a proxy for both procedural rigor and operational capacity.

*Screen-failure rates*. The proportion of participants who fail screening is a long-recognized but underutilized signal. Excessively high failure rates may indicate poor pre-screening practices or overly restrictive interpretation of criteria, while abnormally low rates may signal leniency or misapplication of thresholds. Tracking the distribution of screen-failure rates across sites, and over time, allows monitoring teams to identify centers that deviate substantially from expected benchmarks.

*Screening duration and completion times*. The time required to complete full screening-from consent to final eligibility determination-is another critical metric. Prolonged durations may arise from repeating laboratory tests, waiting for imaging, or delayed adjudication of comorbidities. Such delays are not only operationally inefficient but can also bias trial outcomes if patients’ disease status changes during the waiting period. Monitoring the distribution of completion times provides insight into whether sites are consistently adhering to protocol-defined window periods.

*Window-period adherence*. Most protocols specify maximum allowable intervals between screening assessments and randomization (e.g., laboratory results within 14 days). Deviations from these windows can compromise eligibility validity, especially for dynamic measures such as hematology or liver function. Sites with recurrent window violations introduce systematic risk, and distributional analysis of adherence across sites highlights centers requiring corrective action.

*Impact of disease severit*y. Disease severity at enrollment often interacts with screening dynamics. For example, patients with advanced disease may experience rapid progression during screening, leading to higher failure rates or protocol deviations. Conversely, milder cases may complete screening without delay but skew trial generalizability. Incorporating disease-severity distributions into monitoring allows trial teams to detect whether certain sites disproportionately recruit at one end of the spectrum, creating imbalances in outcome occurrence and event timing.

*Outcome occurrence patterns*. Early occurrence (or delay) of key trial outcomes, such as adverse events or disease progression, can also reflect upstream enrollment practices. Sites enrolling disproportionately severe patients may report outcomes earlier than expected, whereas overly selective sites may delay outcome accumulation. Monitoring the distribution of outcome timing across sites thus provides an indirect check on whether enrollment practices are biasing the trial’s event profile. However, any indicator that makes use of post-randomization outcome information is inherently sensitive to blinding considerations. In practice, such outcome-based monitoring should either rely on pooled, non–arm-specific summaries that do not reveal treatment allocation, or be restricted to an independent data monitoring body (e.g., DSMB/IDMC) that is already charged with reviewing unblinded data. Accordingly, our core enrollment-centric RBM framework can be implemented entirely on pre-randomization baseline and screening/process variables, with outcome occurrence patterns treated as an optional extension in trials that incorporate formal interim monitoring.

### Proposed Enrollment-Integrity Indicators

**Fig 1:**
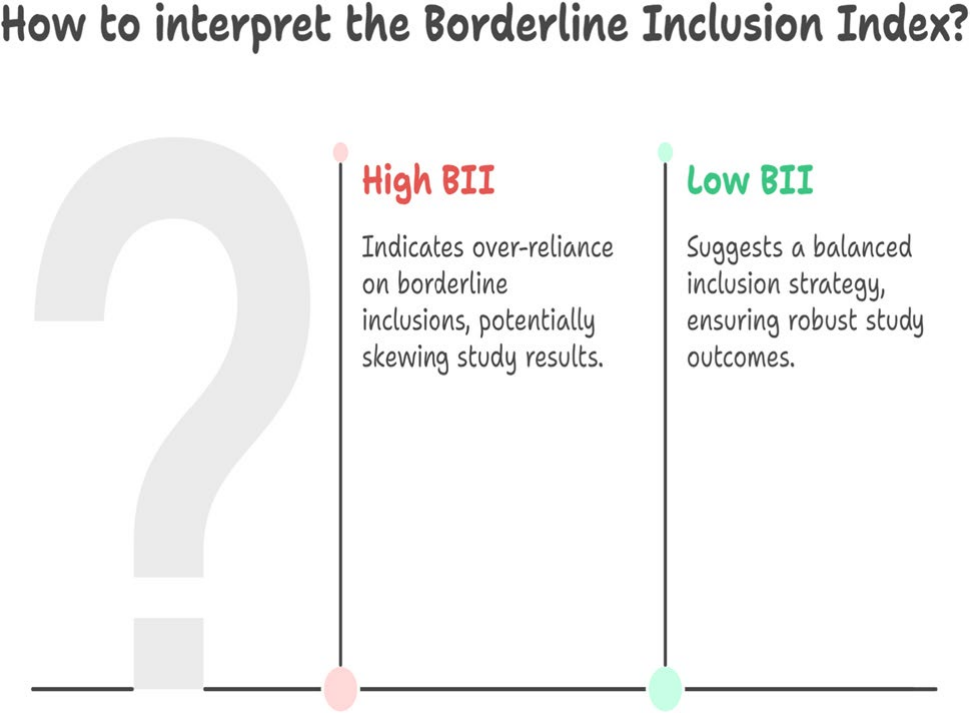
Fig. 1 illustrates how the borderline Inclusion Index (BII) quantifies the proportion of borderline eligibility cases, helping detect sites that may be applying thresholds leniently or inconsistently

To operationalize these insights, we extend RBM with four complementary indicators. The *Borderline Inclusion Index (BII)* quantifies the fraction of participants at each site whose eligibility values fall within a narrow, clinically meaningful band around thresholds, detecting over-reliance on borderline inclusions. The *Eligibility Distribution Divergence (EDD)* measures the degree to which site-level distributions of eligibility variables deviate from the overall trial distribution, adjusting for sample size through hierarchical shrinkage. The *Screen-Failure Pattern Shift (SFPS)* uses change-detection methods applied to reasons for screen failures to capture evolving site practices across time. Finally, the *Consistency with Modernized Guidance (CMG)* assesses whether sites disproportionately exclude patients based on outdated or unnecessarily restrictive criteria (e.g., controlled HIV infection, stable brain metastases), thereby aligning site practices with contemporary regulatory expectations [14, 15]. For CMG, exclusion rates for specific modernized criteria are compared against trial-wide benchmarks or expected rates derived from literature (Fig. 1)

**Fig. 2:**
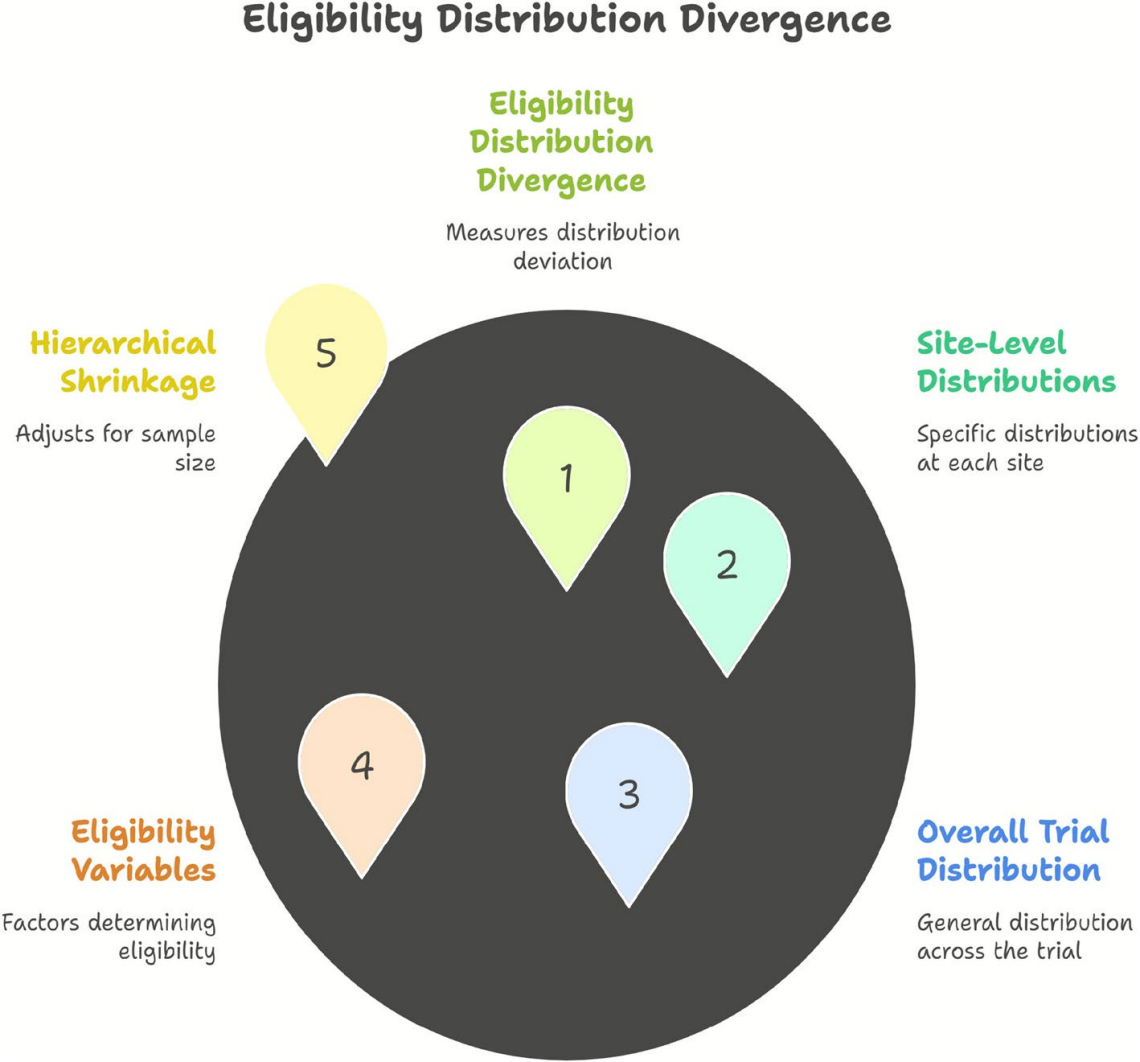
Eligibility distribution divergence (EDD). This indicator measures how site-level eligibility variable distributions deviate from the overall trial distribution, while adjusting for sample size using hierarchical shrinkage

The *Eligibility Distribution Divergence (EDD)* (Fig. 2) captures distributional imbalances in eligibility criteria across sites. It quantifies the extent to which site-level distributions differ from the overall trial distribution. Importantly, it incorporates *hierarchical shrinkage* to adjust for variability in site sample sizes, reducing false positives from small sites. By examining eligibility variables, site-specific distributions, and the trial-wide reference, EDD provides a rigorous metric for detecting systematic enrollment biases and site-level deviations that may impact trial generalizability.

**Fig. 3:**
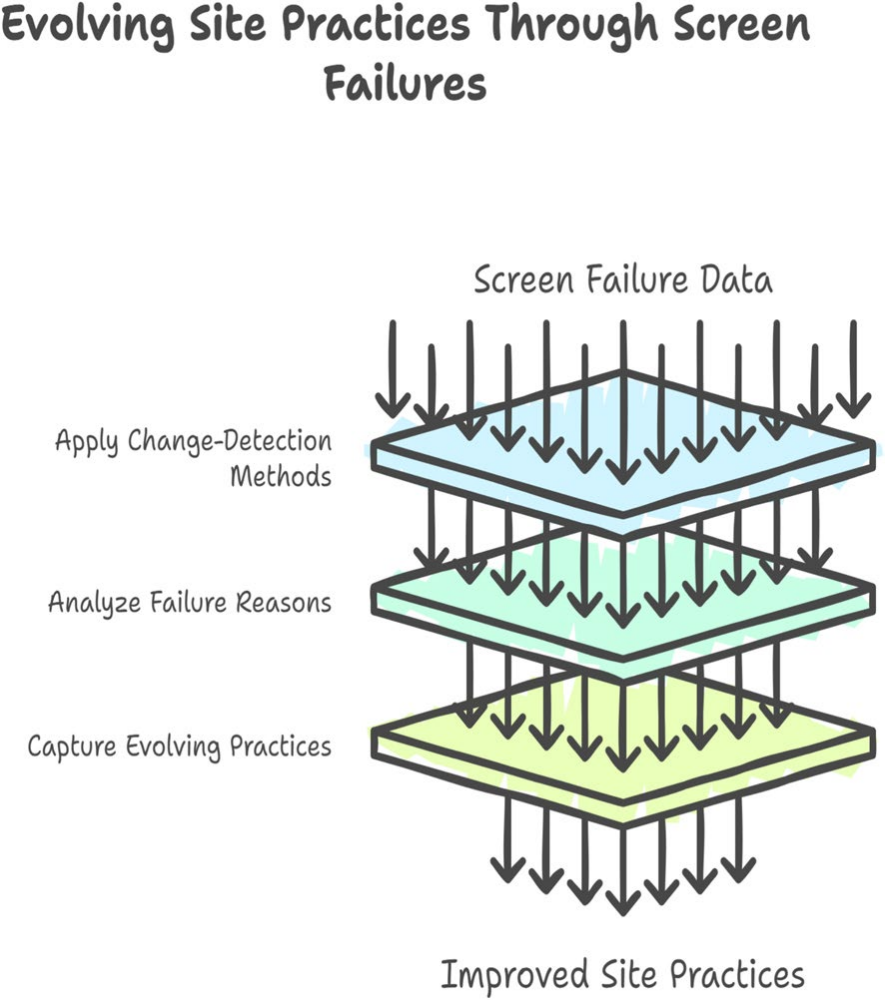
Screen-failure pattern shift (SFPS). This indicator applies change-detection methods to screen-failure data, analyzing evolving reasons for ineligibility across time to identify shifts in site practices

The *Screen-Failure Pattern Shift (SFPS)* (Fig. 3) tracks evolving enrollment behaviors by analyzing reasons for screen failures over time. Change-detection methods are applied to site-level screen-failure data, enabling detection of subtle but systematic shifts in how sites apply eligibility criteria. By examining changes in failure reasons, SFPS captures evolving site practices, allowing early identification of deviations from protocol intent or inconsistent application of inclusion and exclusion rules. This temporal perspective provides an added layer of oversight beyond static enrollment summaries, ensuring that sites remain aligned with the trial’s scientific and ethical framework.

**Fig. 4:**
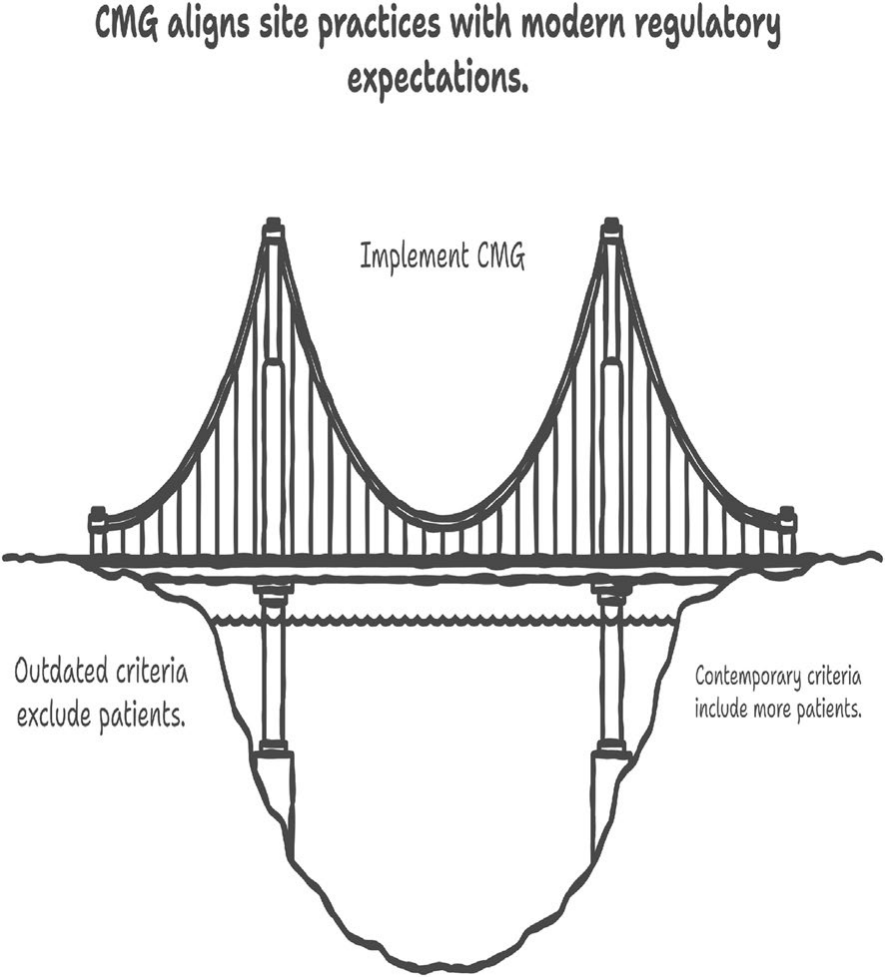
Consistency with modernized guidance (CMG). This indicator evaluates whether sites are disproportionately excluding patients based on outdated or unnecessarily restrictive criteria, bridging site practices with contemporary regulatory expectations

To operationalize these insights, we extend Risk-Based Monitoring (RBM) with four complementary indicators. The *Borderline Inclusion Index (BII)* quantifies the fraction of participants at each site whose eligibility values fall within a narrow, clinically meaningful band around thresholds, detecting over-reliance on borderline inclusions (Fig. 1). The *Eligibility Distribution Divergence (EDD)* measures the degree to which site-level distributions of eligibility variables deviate from the overall trial distribution, adjusting for sample size through hierarchical shrinkage. The *Screen-Failure Pattern Shift (SFPS)* applies change-detection methods to reasons for screen failures, capturing evolving site practices across time. Finally, the *Consistency with Modernized Guidance (CMG)* assesses whether sites disproportionately exclude patients based on outdated or unnecessarily restrictive criteria (e.g., controlled HIV infection, stable brain metastases), aligning site practices with contemporary regulatory expectations [14, 15]. For CMG, exclusion rates for specific modernized criteria are compared against trial-wide benchmarks or expected rates derived from literature (Fig. 4).

The *Consistency with Modernized Guidance (CMG)* (Fig. 3) assesses whether trial sites disproportionately exclude participants on the basis of outdated or unnecessarily restrictive eligibility criteria. Examples include exclusion of patients with controlled HIV infection or stable brain metastases, conditions for which contemporary guidance supports inclusion. By comparing exclusion rates against trial-wide benchmarks or literature-derived expectations, CMG highlights whether site practices are aligned with evolving regulatory recommendations [14, 15]. In doing so, CMG functions as a “bridge” between traditional exclusionary practices and modernized, inclusive enrollment strategies that improve generalizability and equity of clinical trials.

### Integration into Risk-Based Monitoring

These indicators are designed to complement, not replace, existing operational key risk indicators (KRIs). We propose integrating them into centralized RBM dashboards, where each site receives an enrollment-integrity profile in parallel with conventional operational indicators. Escalation follows a dual-key principle: an enrollment-based anomaly is acted upon only when corroborated by an operational signal, reducing false positives and ensuring proportionate responses. For example, a site with unusually high BII and simultaneous increases in protocol deviations would warrant targeted source data verification or retraining. By addressing risks across pre-screening, screening, post-screening, and window-period adherence, and by analyzing distributions rather than isolated thresholds, this framework fills a major gap in RBM and provides sponsors with an evidence-based, audit-ready system for safeguarding trial validity at the point of enrollment.

Together, these metrics-screening velocity, screen-failure rates, screening completion times, window adherence, disease-severity balance, and outcome occurrence patterns-form a holistic view of enrollment quality. Importantly, each metric is meaningful not only in isolation but also in its distribution across sites. A site performing within expected ranges on one metric but persistently outlying on others may represent a latent risk requiring intervention. Embedding these operational metrics alongside eligibility-based indicators (BII, EDD, SFPS, CMG) creates a comprehensive enrollment-monitoring framework. Such integration ensures that RBM addresses not only who enters the trial but also how the process of enrollment unfolds in practice, thereby safeguarding both validity and efficiency.

## Composite Scoring and Bayesian Integration

### Notation and Alignment of Indicators

Let $s=1,\dots ,S$ index sites and $k=1,\dots ,K$ index enrollment/process indicators that may be proportions (e.g., screen failures), times (e.g., screening duration), counts (e.g., window violations), continuous distances (e.g., EDD), or change-point probabilities (e.g., SFPS). Denote the raw metric for site *s* and indicator *k* by $y_{ks}$ with sampling information (e.g., denominators) $n_{ks}$ where relevant.

Each metric is first *direction-aligned* so that larger values indicate higher risk:

$$ \tilde{y}_{ks} = {\left\{ \begin{array}{ll} +y_{ks}, & \text {if higher means higher risk } \\ -y_{ks}, & \text {if lower means higher risk } \end{array}\right. } $$

We then apply an indicator-specific monotone transform $f_k(\cdot )$ to place indicators on a comparable, approximately additive scale:

1 $$\begin{aligned} z_{ks}&= \frac{f_k(\tilde{y}_{ks}) - \textrm{median}_s\{f_k(\tilde{y}_{ks})\}}{1.4826\,\textrm{MAD}_s\{f_k(\tilde{y}_{ks})\}} \end{aligned}$$

2 $$\begin{aligned} f_k(\cdot )&\in \{\log (\cdot ),\;\textrm{logit}(\cdot ),\;\log (1+\cdot ),\;\textrm{id}\} \end{aligned}$$

where MAD denotes the median absolute deviation. Typical choices: $\textrm{logit}$ for proportions (e.g., screen-failure rate, BII, CMG), $\log$ for positive times (e.g., screening duration) and distances (EDD), identity for standardized scores, and $\textrm{logit}$ for change-point posterior probabilities (SFPS). The result $z_{ks}$ is a robust, unitless “risk z-score.”

### Frequentist Composite Score and Alternatives

A simple composite site score is a convex combination

$$ S_s \;=\; \sum _{k=1}^{K} w_k\,z_{ks}, \qquad w_k \ge 0,\quad \sum _{k=1}^{K} w_k = 1. $$

This yields a *linear utility* (“OR/AND neutral”). Two useful generalizations capture different escalation logics:

$$\begin{aligned} \text {Softmax (``OR-leaning'')}: \end{aligned}$$

$$ S^{(\lambda )}_s = \frac{1}{\lambda }\log \!\left( \sum _{k=1}^{K} \exp \{\lambda \,w_k z_{ks}\}\right) , \qquad \lambda > 0 $$

$$\begin{aligned} \text {Geometric mean (``AND-leaning'')}: \end{aligned}$$

$$ G_s = \prod _{k=1}^{K} (c + z_{ks})^{w_k} - c, \qquad c > \max _s\{-\min _k z_{ks}\}. $$

Softmax emphasizes any single very high-risk signal (useful for safety); the geometric mean down-weights sites that are not consistently risky across indicators (useful for data integrity).

Weighting mechanisms. (i) Expert/CTQ weights: set $w_k \propto$ critical-to-quality mapping (e.g., safety vs. data integrity vs. generalizability). (ii) Data-driven (unsupervised): entropy weights that reward indicators with higher cross-site dispersion; or principal-component weights from $\textrm{cov}(z_{\cdot k})$. (iii) Supervised: if historical audits label problem sites, estimate *w* by maximizing the AUC or a logistic loss predicting problems from $z_{ks}$ (with non-negativity and simplex constraints). In practice, we recommend that the primary weighting scheme be pre-specified in the monitoring plan (e.g., based on CTQ considerations and historical experience), with only a limited set of sensitivity analyses, to avoid ad hoc reweighting after inspection of trial data.

Worked toy example (deterministic). Suppose $$K{=}4$$ indicators with robust scores at a site: $$(z_{1s},z_{2s},z_{3s},z_{4s})$$$$=(1.0,\,0.0,\,2.0,\,-1.0)$$ and equal weights $$w_k=0.25$$. Then

$$ S_s \;=\; 0.25(1.0+0.0+2.0-1.0) \;=\; 0.50. $$

With an “OR-leaning” softmax at $\lambda {=}2$, $S^{(2)}_s \approx 0.88$, exceeding 0.50 because $z_{3s}{=}2.0$ dominates.

### Bayesian Hierarchical Integration of Heterogeneous Indicators

Deterministic $z_{ks}$ ignore sampling uncertainty, which can be substantial for small sites. A Bayesian approach treats each $y_{ks}$ with an appropriate likelihood and *partially pools* site-level parameters (via shrinkage toward trial-wide means), producing posterior distributions for the latent risk contributions.

Likelihoods (examples).

3 $$\begin{aligned} \text {Proportions (e.g., BII, CMG, failure rate):} \nonumber \\ y_{ks} \mid \pi _{ks}&\sim \textrm{Binomial}(n_{ks}, \pi _{ks}) \end{aligned}$$

4 $$\begin{aligned} \text {Times (e.g., screening duration, days):} \nonumber \\ \log y_{ks} \mid \mu _{ks}, \sigma _k^2&\sim \mathcal {N}(\mu _{ks}, \sigma _k^2) \end{aligned}$$

5 $$\begin{aligned} \text {Distances (EDD, positive):} \nonumber \\ \log y_{ks} \mid \mu _{ks}, \tau _k^2&\sim \mathcal {N}(\mu _{ks}, \tau _k^2) \end{aligned}$$

6 $$\begin{aligned} \text {Window violations (counts):} \nonumber \\ y_{ks} \mid \theta _{ks}&\sim \textrm{Poisson}(n_{ks}\,\theta _{ks}) \end{aligned}$$

7 $$\begin{aligned} \text {or} \quad y_{ks}&\sim \textrm{Binomial}(n_{ks}, \theta _{ks}) \end{aligned}$$

8 $$\begin{aligned} \text {Change-point (SFPS):} \nonumber \\ m_{ks} \mid \psi _{ks}&\sim \textrm{Bernoulli}(\psi _{ks}) \end{aligned}$$

9 $$\begin{aligned} m_{ks}&= \textbf{1}\{\text {shift detected}\} \end{aligned}$$

Latent site risk factor. Let $R_s$ be a latent continuous “site risk” that co-varies with multiple indicators via generalized linear mixed models (a one-factor Bayesian model):

10 $$\begin{aligned} \textrm{logit}(\pi _{ks})&= \beta _{k0} + \beta _{k} R_s + \epsilon _{ks}^{(\pi )}, \end{aligned}$$

11 $$\begin{aligned}&\epsilon _{ks}^{(\pi )} \sim \mathcal {N}(0,\sigma _{k\pi }^2) \end{aligned}$$

12 $$\begin{aligned} \mu _{ks}&= \gamma _{k0} + \gamma _k R_s + \epsilon _{ks}^{(\mu )}, \end{aligned}$$

13 $$\begin{aligned}&\epsilon _{ks}^{(\mu )} \sim \mathcal {N}(0,\sigma _{k\mu }^2) \end{aligned}$$

14 $$\begin{aligned} \textrm{logit}(\psi _{ks})&= \delta _{k0} + \delta _k R_s \end{aligned}$$

15 $$\begin{aligned} R_s&\sim \mathcal {N}(0,1) \end{aligned}$$

This yields cross-indicator *partial pooling* and embeds dependence: if a site is risky on one indicator, $R_s$ raises expectations for others in a data-driven way.

Priors. Weakly informative priors stabilize estimation:

16 $$\begin{aligned} \beta _{k0},\;\gamma _{k0},\;\delta _{k0}&\sim \mathcal {N}(0,5^2) \end{aligned}$$

17 $$\begin{aligned} \beta _k,\;\gamma _k,\;\delta _k&\sim \mathcal {N}^+(0,1^2) \quad \end{aligned}$$

18 $$\begin{aligned} \text {(half-normal, nonnegative loadings)} \end{aligned}$$

19 $$\begin{aligned} \sigma _{k\pi },\;\sigma _{k\mu }&\sim \textrm{HalfCauchy}(0,1) \end{aligned}$$

Nonnegativity encodes the design that higher $R_s$ implies higher risk across indicators.

Posterior composite scores. For each site, define risk-aligned latent contributions

$$ Z_{ks}^\star \equiv {\left\{ \begin{array}{ll} \textrm{logit}(\pi _{ks}) & \text {for proportions}, \\ \mu _{ks} & \text {for log-times or log-distances}, \\ \textrm{logit}(\psi _{ks}) & \text {for change-point probability}, \end{array}\right. } $$

and a posterior composite

20 $$\begin{aligned} S_s^{\text {Bayes}}&= \sum _{k=1}^{K} w_k\,\frac{Z_{ks}^\star - \tilde{\mu }_k}{\tilde{\sigma }_k} \end{aligned}$$

21 $$\begin{aligned} \tilde{\mu }_k,\;\tilde{\sigma }_k&\text { taken as trial-level posteriors (robust).} \end{aligned}$$

Crucially, $S_s^{\text {Bayes}}$ is a *distribution*, not a point: we report its posterior mean and credible interval, and use it for probability-based ranking.

Bayesian weighting of indicators. Instead of fixing *w*, place a simplex prior

$$ \textbf{w}=(w_1,\dots ,w_K) \sim \textrm{Dirichlet}(\alpha _1,\dots ,\alpha _K), $$

where $\alpha _k$ reflects CTQ criticality (e.g., larger for safety-critical indicators). If historical labeled data exist (audit outcomes), one can form a supervised likelihood for $\textbf{w}$ by linking $S_s^{\text {Bayes}}$ to audit outcomes via a probit/logit layer, thereby updating $\textbf{w}$ to emphasize historically predictive indicators. A sparsity-friendly alternative is a spike-and-slab on $w_k$ or a Dirichlet-*Laplace* prior.

### Posterior ranking, flagging, and uncertainty

Given posterior draws $\{S_{s}^{(m)}\}_{m=1}^{M}$ or $\{R_{s}^{(m)}\}_{m=1}^{M}$:

22 $$\begin{aligned} \widehat{\Pr }\{S_s> \tau \mid \text {data}\}&= \frac{1}{M}\sum _{m=1}^{M} \textbf{1}\{S_s^{(m)} > \tau \}, \end{aligned}$$

23 $$\begin{aligned} \widehat{\Pr }\{s \in \text {Top-}K\}&= \frac{1}{M}\sum _{m=1}^{M} \textbf{1}\{\textrm{rank}(S_{\cdot }^{(m)}) \le K\}. \end{aligned}$$

Importantly, the framework does not assume that sites are uniformly distributed in terms of patient volume or accrual dynamics. Proportion-based indicators such as BII, screen-failure rates, and window adherence are modeled with denominators $n_{ks}$, so that small or newly opened sites naturally yield wide posteriors and contribute more cautiously to the composite score. Distance-based indicators such as EDD are computed using sample-size–dependent two-sample distances and then standardized across sites using robust (median/MAD) scaling. Together with the use of posterior distributions rather than point estimates, this partial pooling helps prevent sites from being flagged solely because of low accrual or early calendar time, and ensures that apparent anomalies are interpreted relative to both uncertainty and expected variability.

Decision rule (dual-key with Bayesian control). Flag site *s* if

24 $$\begin{aligned} \Pr \{S_s> \tau \mid \text {data}\}&> p^\star \end{aligned}$$

25 $$\begin{aligned} \Pr \{Z_{k^\dagger s}^\star> \tau _{k^\dagger } \mid \text {data}\}&> p^\star \end{aligned}$$

where $k^\dagger$ is an operational corroborator (e.g., protocol-deviation rate). To control false discovery, choose $\tau ,p^\star$ by bounding the Bayesian false discovery rate

26 $$\begin{aligned} \pi _{1s} \mid y_{1s}, n_{1s}&\sim \textrm{Beta}\!\left( a_1+y_{1s},\, b_1+n_{1s}-y_{1s}\right) \end{aligned}$$

27 $$\begin{aligned} Z_{1s}^\star&= \textrm{logit}\!\left( \pi _{1s}\right) \end{aligned}$$

where $\mathcal {F}$ is the flagged set and *q* is a desired level (e.g., 10%).

### Worked Bayesian Illustrations

(A) Borderline Inclusion Index (proportion). At site *s*, $y_{1s}$ borderline inclusions out of $n_{1s}$ enrolled. With $\pi _{1s}\sim \textrm{Beta}(a_1,b_1)$,

28 $$\begin{aligned} \pi _{1s}\mid y_{1s},n_{1s}&\sim \textrm{Beta}\!\left( a_1+y_{1s},\, b_1+n_{1s}-y_{1s}\right) \end{aligned}$$

29 $$\begin{aligned} Z_{1s}^\star&= \textrm{logit}\!\left( \pi _{1s}\right) \end{aligned}$$

For small $n_{1s}$ the posterior is wide, correctly widening the credible band for $S_s^{\text {Bayes}}$.

(B) Screening duration (log-normal). Let $t_{2s}$ be the site median duration; model $\log t_{2s}\mid \mu _{2s},\sigma _2^2 \sim \mathcal {N}(\mu _{2s},\sigma _2^2)$ with $\mu _{2s}\sim \mathcal {N}(\gamma _{20}+\gamma _2 R_s,\sigma _{2\mu }^2)$. Then $Z_{2s}^\star =\mu _{2s}$ and contributes additively to $S_s^{\text {Bayes}}$ after robust standardization.

(C) EDD (positive distance). Model $$ \log ({\mathrm{EDD}}_{s} ) $$$$ {\mid }\mu _{{3s}} $$$$ \tau _{3}^{2} \sim $$$${\mathcal{N}} $$$$ (\mu _{{3s}} ,\tau _{3}^{2} ) $$ with $$ \mu _{{3s}} = $$$$\gamma _{{30}} $$$$ + \gamma _{3} $$$$ R_{s} + _{{3s}} $$; again $$ Z_{{3s}}^{{ \star }} $$$$ = \mu _{{3s}} $$.

(D) SFPS (change-point). If $$m_{4s}\in \{0,1\}$$ denotes a detected shift, use $$ \Pr (m_{{4s}} = 1) $$$$ = \psi _{{4s}} $$ with $$ {\mathrm{logit}}(\psi _{{4s}} ) $$$$ = \delta _{{40}} + \delta _{4} R_{s} $$; then $$Z_{4s}^\star =\textrm{logit}(\psi _{4s})$$.

### Handling Window Periods, Severity Mix, and Missingness

Window adherence can be modeled as $$ y_{{5s}} \sim $$$$ {\mathrm{Binomial}} $$$$ (n_{{5s}} ,\theta _{{5s}} ) $$, $$\textrm{logit}(\theta _{5s})=\beta _{50}+\beta _5 R_s$$. Severity mix across categories (e.g., ECOG levels) can be captured by a logistic-normal multinomial: $$\boldsymbol{\vartheta }_s \sim \textrm{LogitNormal}(\textbf{A}\,R_s,\Sigma )$$; a divergence (e.g., KL or energy distance) to the trial-wide mix then feeds an EDD-type indicator. Missingness in $$y_{ks}$$ can be accommodated by data augmentation (e.g., Beta-Binomial with missing $$n_{ks}$$ segments) or by modeling missing-not-at-random mechanisms tied to $$R_s$$, thereby preventing optimistic bias for sites with sparse reporting.

### Reporting and Visualization

For each site, report $\mathbb {E}[S_s^{\text {Bayes}}\mid \text {data}]$ with 95% credible intervals (CrI), $\Pr \{S_s>\tau \mid \text {data}\}$, and $\Pr \{\text {Top-}K\}$. Provide tornado plots of the posterior contributions $w_k\,Z_{ks}^\star$ to explain which indicators drive the risk and how uncertainty from small denominators propagates into $S_s^{\text {Bayes}}$.

### Practical Guidance on Priors and Computation

Set Dirichlet weights with $\alpha _k$ proportional to CTQ importance (e.g., safety-critical $\alpha _k{=}4$, data-quality $\alpha _k{=}2$, generalizability $\alpha _k{=}1$). Use weakly informative Normal/Half-Cauchy priors; fit with Hamiltonian Monte Carlo or variational Bayes. In large trials, a two-step approximation works well: (1) site-level posteriors for $Z_{ks}^\star$ in parallel; (2) combine via draws of $\textbf{w}$ to obtain $S_s^{\text {Bayes}}$ and ranks with uncertainty.

### Interpretation

The composite $S_s^{\text {Bayes}}$ provides (i) a single, transparent summary for governance decisions and (ii) full uncertainty quantification for proportionate escalation. Because it integrates process speed (screening velocity, completion times, window adherence), quality (screen-failure rates, SFPS), eligibility integrity (BII, EDD, CMG), and severity/outcome timing, it acts as a scientifically grounded, audit-ready basis for risk-based monitoring and site ranking.

## Interactive Application for Enrollment-Centric RBM

We developed an interactive Shiny application that operationalizes the proposed enrollment-centric risk-based monitoring (RBM) framework. The app ingests either simulated or user-provided site-level data, constructs simple Bayesian-style posteriors for key enrollment and process metrics, standardizes these signals to a common risk scale, and aggregates them into a composite site risk score *S* with full uncertainty. Sites are then ranked by posterior summaries, and flags are issued using a dual-key decision rule. The application is designed for routine RBM cycles, emphasizing transparency, auditability, and alignment with quality-by-design principles.

### Inputs and Data Model

Data sources. The app supports:

1. *Simulation mode*, which generates a multicenter dataset with realistic heterogeneity in screening, eligibility, and process speed; and
2. Upload mode, which validates and merges three CSV files:    1) site_agg.csv: site, n_screen, n_enrolled, y_fail, y_window, y_bii, n_bii, m_shift;    2) labs.csv: long-form records (site, lab_value) for each enrolled participant;    3) times.csv: long-form records (site, screening_time) for each screened participant.

Shape checks ensure consistency (e.g., the number of lab rows must equal n_enrolled at a site), preventing silent misalignment.

### Monitored Metrics

The app computes six complementary indicators that jointly reflect enrollment integrity, process quality, and behavioral drift:

1. Borderline Inclusion Index (BII): fraction of enrolled participants with baseline eligibility value in a narrow window $[\tau -\delta ,\,\tau +\delta ]$ around the prespecified threshold $\tau$.
2. Screen-failure rate: $y_{\textrm{fail},s}/n_{\textrm{screen},s}$ at site *s*.
3. Window adherence (risk-aligned): $y_{\textrm{win},s}/n_{\textrm{enr},s}$, flipped so that lower adherence implies higher risk.
4. Screening duration: median of per-screened participant screening times on the log scale.
5. Eligibility Distribution Divergence (EDD): two-sample Kolmogorov–Smirnov distance $D_s$ between a site’s lab distribution and the pooled distribution of *other* sites.
6. Screen-Failure Pattern Shift (SFPS): probability that the composition of screen-failure reasons has materially changed (a drift proxy) (Table 1).

**Table 1:** Summary of monitored indicators in the shiny application

Indicator	BII
Definition	Fraction in borderline window
Likelihood Model	Binomial

Indicator	Screen-failure rate
Definition	Failures / screened
Likelihood Model	Binomial

Indicator	Window adherence
Definition	Violations / enrolled (negated)
Likelihood Model	Binomial

Indicator	Screening duration
Definition	Log median time
Likelihood Model	Normal

Indicator	EDD
Definition	KS distance to others
Likelihood Model	Log-normal

Indicator	SFPS
Definition	Drift detection probability
Likelihood Model	Bernoulli

### Posterior Construction (Stage 1)

To quantify uncertainty, each metric is mapped to a simple likelihood and posterior:

Proportions (BII, screen failure, window adherence). For site *s* and metric *k* with $y_{ks}$ successes out of $n_{ks}$,

$$ \begin{aligned} \pi _{ks} \mid y_{ks}, n_{ks}&\sim \textrm{Beta}\bigl (1+y_{ks},\,1+n_{ks}-y_{ks}\bigr ), \\ z^{\star }_{ks}&= \textrm{logit}\!\left( \pi _{ks}\right) . \end{aligned} $$

where the $\textrm{Beta}(1,1)$ prior provides weak regularization and avoids degenerate 0/1 issues.

Screening duration (log scale). Let $t_{sj}$ be screening times for screened participants at site *s*; we bootstrap the median of $\{\log t_{sj}\}$ to obtain draws $z^{\star }_{\textrm{dur},s}$.

EDD (KS distance). We form $D_s$ by comparing the site’s lab values with the pooled values of *other* sites, then bootstrap $D_s$ by resampling both samples; the transformed draws are $z^{\star }_{\textrm{edd},s}=\log \{\max (D_s,10^{-6})\}$ for stability.

SFPS (drift). Given a binary drift flag $m_s\in \{0,1\}$,

$$ \begin{aligned} \psi _s \mid m_s&\sim \textrm{Beta}\bigl (1+m_s,\,1+1-m_s\bigr ), \\ z^{\star }_{\textrm{sfps},s}&= \textrm{logit}\!\left( \psi _s\right) . \end{aligned} $$

Risk alignment. All metrics are oriented so that larger values imply higher risk; in particular, window adherence is negated on the logit scale.

### Robust Standardization (Stage 2)

Because metrics are on disparate scales, each is standardized to robust *z* units per draw:

1. compute site-level posterior means as anchors for each metric;
2. across sites, take the median as *center* and the MAD as *scale* (fallback to SD if MAD$=0$);
3. for each draw, set $z_{ks,\textrm{std}}^{(m)}=\{z_{ks}^{\star (m)}-\textrm{center}_k\}/\textrm{scale}_k$.

This yields unitless, comparable risk scores across all metrics and sites.

### Composite Score and Weights (Stage 3)

For site *s* and draw *m*, the composite score is

30 $$\begin{aligned} S_s^{(m)}=\sum _{k=1}^{K} w_k\, z_{ks,\textrm{std}}^{(m)}, \qquad w_k\ge 0,\quad \sum _{k=1}^{K} w_k=1, \end{aligned}$$

with $K=6$ in the current implementation. Weights $(w_k)$ are user-specified in the UI (auto-normalized), allowing alignment with critical-to-quality (CTQ) priorities (e.g., emphasizing EDD and BII for eligibility integrity versus duration and window adherence for process discipline). The posterior of $S_s$ is summarized by $\mathbb {E}[S_s]$, a 95% credible interval (CrI), $\Pr (S_s>\tau )$ for a user-selected threshold $\tau$, and $\Pr (\text {Top-}K)$ based on per-draw ranks.

### Ranking, Thresholding, and Flagging

Ranking. Sites are ordered by posterior mean $\mathbb {E}[S_s]$, with uncertainty conveyed via CrIs and $\Pr (\text {Top-}K)$.

Thresholding. For a decision threshold $\tau$, the exceedance probability is

$$ \Pr (S_s>\tau \mid \text {data}) \approx M^{-1}\sum _{m=1}^{M}\textbf{1}\{S_s^{(m)}>\tau \}. $$

Dual-key flag (example). To guard against single-metric anomalies, the app implements a dual-key rule: flag site *s* if

$$ \Pr (S_s>\tau \mid \text {data})>p^\star \quad \text {and}\quad \overline{z}_{\textrm{fail},s,\textrm{std}} > c, $$

where $\overline{z}_{\textrm{fail},s,\textrm{std}}$ is the posterior mean standardized screen-failure component, $p^\star$ is a confidence level (e.g., 0.8), and *c* a corroborator cut-point (default 0.5). Alternative corroborators (e.g., protocol deviations) can be substituted.

### Visual Analytics and Decision Support

Forest plot. Posterior mean *S* with 95% CrI per site (*who* is high risk and *how* uncertain the ordering is).

Heatmap. Weighted contributions (diverging scale centered at 0) by component and site (*which components* drive risk patterns across sites).

Drivers (small multiples). For selected sites, diverging bars show component-level contributions ordered by absolute impact, with numeric labels. This directly informs targeted corrective actions (e.g., elevated BII and EDD plus prolonged duration suggests threshold interpretation issues and operational bottlenecks).

### Interpretation Guidance

Clear signal; actionable. A site with $\mathbb {E}[S_s]$ high, narrow CrI, and coherent drivers (e.g., large EDD and BII plus long duration) warrants targeted intervention: eligibility retraining, assay checks, scheduling support, and possibly focused SDV on borderline cases.

Borderline; monitor. A site with moderate *S* and wide CrI or mixed drivers is best treated with feedback and re-evaluation in the next monitoring cycle.

Low risk. A site with negative *S* and stable components is a candidate for positive deviance review (sharing good practices).

### Design Choices and Guardrails

- Uncertainty first: Every metric produces a posterior distribution; small sites are not over-penalized.
- Robust scaling: Median/MAD protects against outliers; SD fallback ensures numerical stability.
- EDD pooling: The focal site is excluded from the pool, avoiding self-comparison bias.
- \Dual-key triggers: Combining an enrollment-centric anomaly with an operational corroborator reduces false positives and aligns actions with CTQs.

### User Controls and Sensitivity

The UI exposes (i) number of posterior draws *M* (speed vs. precision), (ii) CTQ-aligned weights $(w_k)$ (auto-normalized), (iii) threshold $\tau$ and Top-*K*, and (iv) simulation knobs (number of sites, screening range, threshold $\tau _{\textrm{lab}}$). Sensitivity analyses—varying $(w_k)$, $\tau$, and bootstrap sizes—are recommended and can be exported for documentation.

### Governance Workflow

A typical RBM cycle comprises: (1) ingest latest data; (2) regenerate posteriors, rankings, flags; (3) triage with the forest plot; (4) diagnose drivers per site and record decisions/CAPAs; (5) confirm signal resolution next cycle (decrease in *S*; normalization of drivers). All tables and matrices are downloadable for audit trails.

### Limitations and Extensions

The current implementation uses conjugate Beta posteriors for proportions and bootstraps for log medians and KS distances to balance transparency and speed. For greater tail sensitivity, EDD can be extended to energy distance or Anderson–Darling. SFPS can be enriched with CUSUM/Bai–Perron time-series models to produce a continuous drift probability. A joint hierarchical latent-factor model that links all metrics through a site-level $R_s$ (with a Dirichlet prior on weights) can be implemented in Stan; our modular design allows swapping Stage 1 posteriors for HMC draws without changing downstream logic.

### Reproducibility and Auditability

Templates ensure consistent shapes and semantics; downloads (site_summary, flags, component_means, posterior_draws) preserve the full analysis state. Simulation mode uses deterministic seeds for reproducibility. All parameters and decisions can be recorded within RBM meeting minutes for regulatory inspection.

### Summary

The application translates the proposed enrollment-centric RBM methodology into an operational, audit-ready tool. It integrates uncertainty-aware indicators (BII, EDD, SFPS, window adherence, screening duration, screen-failure) into a single interpretable score *S* with posterior ranking and explicit drivers, enabling proportionate, data-driven monitoring interventions at the earliest stage of trial conduct.

### Web Application Deployment

To facilitate interactive exploration of the proposed risk-based monitoring (RBM) composite score framework, we deployed a web application built using the R programming language (version 4.x) together with the shiny and flexdashboard packages. The application is hosted on the shinyapps.io platform and is publicly accessible at https://atanu.shinyapps.io/rbm-composite-score/.

The dashboard allows users to either simulate multi-site clinical trial data under user-specified settings or upload site-level input files consisting of (i) aggregated screening and enrollment metrics. In its current implementation, the EDD and BII indicators accept one laboratory-based eligibility variable at a time, which is typical in threshold-driven inclusion criteria (e.g., creatinine clearance or bilirubin). However, the data structure and code are modular, allowing users to upload multiple eligibility variables in long format and compute indicator-specific EDD or BII values iteratively or in parallel. An extended version of the app-with support for multiple simultaneous lab-based eligibility variables-is in development and will be released as an update.

The CMG indicator is implemented through exclusion-rate data (e.g., controlled HIV, stable brain metastasis, organ dysfunction), which currently enters the Shiny app under user-defined exclusion flags. In the revised app (forthcoming), CMG will be surfaced as a separate input field and presented as a stand-alone indicator in the heatmap and composite risk score dashboard to improve transparency and alignment with the manuscript.

(ii) patient-level laboratory values, and (iii) screening time distributions. Once the data are supplied, the application computes Bayesian-like posterior distributions for each monitoring metric, standardizes them, and combines them into a composite site-level risk score.

Key outputs are organized into multiple tabs, including: (i) a forest plot of posterior means with 95% credible intervals for the composite score, (ii) heatmaps and contribution bar charts that visualize the drivers of site-level risk, and (iii) a flagging module that highlights sites with elevated probability of exceeding a prespecified risk threshold. Additional tabs provide guidance on data structure, downloadable templates, and explanatory text designed for non-technical users. The platform also supports export of numerical summaries and graphics to enable downstream reporting or integration with trial monitoring workflows. To encourage adoption and allow interested groups to adapt the framework to their own monitoring environments, we make the underlying R code and example input templates available alongside the deployed application (via supplementary materials and a public version-controlled repository).

This deployment demonstrates the feasibility of translating advanced statistical monitoring methods into a user-friendly, web-based decision-support tool for clinical trial operations.

## Simulation Study

To evaluate the performance of the proposed enrollment-centric indicators (BII, EDD, SFPS, window adherence, screening duration, and screen-failure rate) and the accompanying composite Bayesian score, we implemented a data-generating process that mimics a multicenter trial with heterogeneous site behaviors. Specifically, we simulated $S=50$ sites with between 20 and 100 screened participants per site, drawing enrollment sizes from a discrete uniform distribution. Each participant was assigned a laboratory-based eligibility value from $\mathcal {N}(80,15)$, with a protocol-defined threshold of 60 mL/min. For 10% of sites (designated as atypical), the site-level mean was shifted by $-12$ units to increase borderline inclusions. Screen-failure rates were generated using site-specific Beta–Binomial distributions with means between 0.10 and 0.40. Screening durations followed a log-normal distribution ($\mu =7$ days, $\sigma =0.4$), with atypical sites having median durations 30% higher. Window adherence was modeled as $\textrm{Binomial}(n, \theta _s)$ with $\theta _s \in [0.80,0.95]$. SFPS was generated via a Bernoulli process with a site-specific drift probability.

For each simulated trial instance, we computed the six indicators, transformed them into robust *z*-scores, and constructed the composite Bayesian site score using 1000 posterior draws. True atypical sites were known by design, enabling the computation of sensitivity, specificity, false discovery rate (FDR), and ranking accuracy. We repeated the simulation for 500 Monte Carlo replicates. Additional simulation scenarios were explored, including variation in the prevalence of atypical sites, sample size imbalance, time-varying accrual, and threshold misalignment.

To evaluate the framework, we simulated data from a multicenter trial with 50 sites, each enrolling 20–100 participants. Heterogeneity was introduced by varying site-specific thresholds for a key laboratory marker (e.g., creatinine clearance), with some sites shifted toward the cutoff of 60 mL/min. Additional variability was added to screen-failure rates (10–40%), screening durations (log-normal with mean 7 days), and window adherence (80–95%). Atypical sites (10%) had elevated borderline inclusions and distributional shifts.

Across the Monte Carlo replicates, the enrollment-centric composite score demonstrated sensitivity of 0.83, specificity of 0.88, and mean FDR of 0.14 in detecting atypical sites at a standardized risk-threshold of $\tau = 1.5$. Posterior ranking stability was high (Spearman $\rho = 0.91$ across replicates). Notably, EDD and BII provided complementary signals-EDD detecting distributional shifts, and BII detecting excessive borderline admissions. As expected, very small sites generated wider posterior intervals but were seldom flagged unless corroborated by high-risk indicators. A concise summary of results is shown in Table~1.

## Case Illustration

We applied the framework retrospectively to a multicenter oncology trial (n = 1200 participants, 45 sites), using anonymized site-level data from a study that included renal function eligibility (creatinine clearance > 50 mL/min) as a key inclusion criterion. Study data included screening logs, enrollment dates, laboratory eligibility values, window adherence, screen-failure reasons, and protocol deviation summaries. No treatment-arm information was accessed, ensuring full blinding during metric computation.

BII values ranged from 0.05 to 0.32 across sites (median 0.10), with six sites exceeding a BII of 0.25. EDD values (computed as site-vs-pool KS distances) ranged from 0.04 to 0.31 (median 0.12), with four of the same six sites showing large distributional shifts toward the eligibility threshold. Screening duration and window adherence metrics were consistent with operational logs showing delayed laboratory re-checks.

Composite Bayesian risk scores identified the same six sites as high-risk (posterior $P(S_s> 1.5) \ge 0.85$), and these sites corresponded to locations where the sponsor had later issued targeted training or corrective actions. Although formal ground-truth labels were not available, independent review of blinded monitoring notes confirmed that these sites had a recurring pattern of borderline admissions and incomplete verification of protocol-defined laboratory thresholds.

Table X summarizes the observed BII, EDD, and composite $S_s$ values for selected sites, while Supplementary Material S2 provides anonymized visualizations of posterior score distributions, Shiny dashboard screenshots, and full site rankings. Results demonstrate that enrollment-centric indicators can flag subtle but systematic deviations in eligibility interpretation, even when conventional KRIs (e.g., protocol deviations, enrollment rates) remain within expected limits.

Traditional monitoring had not escalated these sites, but our framework highlighted potential risks earlier, prompting focused eligibility documentation audits and training.

## Discussion

Our results add to a maturing body of work showing that centralized, statistics-driven oversight can detect site-level problems earlier and more proportionately than exhaustive on-site verification. Empirical and methodological studies have demonstrated that central statistical monitoring efficiently uncovers anomalous patterns in accrual, outcome reporting, and baseline distributions [5, 6], and that risk-adapted monitoring delivers comparable protection of data integrity at lower cost than extensive SDV [3, 4]. Adoption data from industry surveys further indicate that risk-based approaches are now mainstream across sponsors and phases, with marked year-on-year growth in RBQM components [16]. Within this landscape, our contribution is to foreground *enrollment-integrity* as a primary, quantitative risk signal and to show how distribution-aware indicators can be fused into a single, uncertainty-aware composite for decision-making.

Methodologically, three choices differentiate our approach from prior work (e.g., [6], which focuses on post-enrollment anomalies). First, we treat enrollment as a *distributional* phenomenon rather than a sequence of pass/fail gates. Tools with sensitivity beyond mean shifts—e.g., the energy distance and the *k*-sample Anderson–Darling statistic [17]—are well suited to detecting subtle shape and tail differences across sites. The Eligibility Distribution Divergence (EDD) used here operationalizes this idea by bootstrapping a site-vs-pooled two-sample distance and transforming it onto a stable scale. Second, we model *temporal drift* in screening behavior using change-point logic (our Screen-Failure Pattern Shift, SFPS). Classic sequential methods such as CUSUM [18] and modern multiple-break detection [19] provide principled foundations for detecting abrupt changes in mix or rates with minimal delay. Third, we quantify uncertainty explicitly. Partial pooling and posterior draws ensure that small sites are not over-penalized and that flagging decisions reflect precision as well as point estimates; these choices are consistent with best practice in hierarchical modeling [20, 21].

Viewing risk through the lens of *who gets enrolled* also aligns with parallel efforts in the oncology literature to modernize eligibility to improve representativeness without compromising safety. The ASCO–Friends series shows how criteria around brain metastases, HIV, organ dysfunction, age, and prior malignancy can be rationalized to preserve internal validity while expanding access [22–24]. Our indicators—Borderline Inclusion Index (BII), EDD, and window adherence—translate those qualitative aims into quantitative, auditable signals. Concretely, a site that over-admits borderline cases (high BII), exhibits non-congruent eligibility distributions (high EDD), and struggles with time windows is plausibly selecting a systematically different baseline population; such a pattern has immediate implications for bias, safety, and time-to-event dynamics.

The governance implications are practical. A composite that is interpretable (linear on robust *z* units) and uncertainty-aware (credible intervals; $P(\text {Top-}K)$; $P(S>\tau )$) supports *proportionate* action: informative feedback when signals are weak or mixed; targeted retraining when a small number of components dominate; focused SDV or source checks when enrollment-centric signals corroborate operational anomalies. This dual-key ethos echoes recommendations from integrated RBM frameworks and scoping reviews that advocate combining multiple central signals with clear escalation logic [25–27].

Our framework is most naturally suited to conventional multicenter randomized trials in which detailed baseline eligibility variables, screening logs, and timing information are routinely collected. In very large/simple trials or in some real-world data settings, eligibility information may be sparser or less standardized, and in such cases only a subset of the proposed indicators (e.g., screen-failure patterns or window adherence) may be implementable. Importantly, the incremental burden is primarily computational and analytic: the approach reuses data that are already captured as part of good clinical practice, and is intended to complement rather than replace existing RBM dashboards and processes.

At the same time, although our primary focus is on centrally implemented, blinded RBM workflows that operate on baseline and process data, the same indicators can be embedded within formal interim monitoring for group-sequential, adaptive, or multi-regional trials. In those settings, outcome occurrence patterns and related post-randomization metrics would naturally fall under the remit of an independent monitoring committee, complementing conventional stopping or adaptation boundaries while preserving appropriate firewalls between operational RBM activities and unblinded efficacy/safety review.

The same structure can be extended to multi-regional clinical trials as described in ICH E17: General Principles for Planning and Design of Multi-Regional Clinical Trials and ICH E5(R1): Ethnic Factors in the Acceptability of Foreign Data [28]. In such settings, intrinsic factors (e.g., demographic, genetic, disease biology) and extrinsic factors (e.g., healthcare practices, cultural norms, regional regulatory expectations) may influence how eligibility criteria are interpreted and applied. Embedding region as a higher-level grouping allows sites to be nested within regions, enabling monitoring teams to detect not only anomalous sites, but also systematic regional shifts in eligibility patterns, borderline inclusions, window adherence, or screening failure reasons. This regional dimension supports early detection of effect modifiers and enhances harmonization of enrollment behavior across regions, consistent with the emphasis in ICH E17 and E5(R1) on variability and heterogeneity in treatment-effect estimation across geographic areas. More generally, real-world trials exhibit heterogeneous accrual dynamics and case-mix across sites, and not all cross-site differences in the proposed indicators should be interpreted as performance problems. Sites that open later, serve distinct referral populations, or operate in health systems with different diagnostic pathways may legitimately display different enrollment profiles. In practice, we recommend computing indicators within calendar-time windows or trial epochs, considering stratification or adjustment by region or other site-level covariates when systematic differences are expected, and treating elevated scores as prompts for targeted review rather than automatic evidence of non-compliance. Clinical and operational context thus remains essential for distinguishing true anomalies from expected heterogeneity.

Limitations include assumptions of independence across indicators (potential extensions: multivariate copulas) and computational scalability for very large trials (mitigated by parallelization). The bootstrap-plus-conjugate implementation trades some modeling richness for transparency and speed; distance measures such as KS can be conservative with small samples, though robust standardization helps. SFPS currently uses a binary flag; continuous time-series models could enhance sensitivity. Weights $w_k$ are user-set; learning from audit outcomes or protocol criticality would personalize further. Validation in prospective settings and across therapeutic areas (beyond oncology) is needed. These are tractable extensions within the architecture.

In sum, enrollment-centric, distribution-aware RBM is both principled and actionable. It complements existing KRIs by moving surveillance upstream to the point where many downstream problems originate, and it does so using methods with strong statistical pedigree and growing empirical support [3, 5, 6, 25, 27].

## Conclusion

The proposed framework expands RBM methodology by incorporating enrollment criteria variability into risk assessment. This represents a step toward more proactive, adaptive, and scientifically grounded trial monitoring systems.

## Data Availability

No datasets were generated or analysed during the current study.
